# Seasonal, morphodynamic, and vegetation drivers of ghost crab (*Ocypode quadrata*) populations on subtropical sandy beaches

**DOI:** 10.1007/s10661-026-15876-z

**Published:** 2026-09-04

**Authors:** Julia Gomes do Vale, Samira Chahad-Ehlers, Germano Henrique Costa Barrilli, Diego Ferreira Gomes, Odete Rocha, Joaquim Olinto Branco

**Affiliations:** 1 Postgraduate Programme in Ecology and Natural Resources, Universidade Federal de São Carlos (UFSCar), Rodovia Washington Luís: Km 235, São Carlos, SP CP676 Brazil; 2 Department of Ecology and Evolutionary Biology, Federal University of São Carlos, Rodovia Washington Luís: Km 235, São Carlos, SP Brazil; 3 Department of Genetics and Evolution, Federal University of São Carlos, Rodovia Washington Luís: Km 235, São Carlos, SP Brazil; 4 Postgraduate Programme in Environmental Oceanography (PPGOAM), Centre for Human and Natural Sciences, Federal University of Espírito Santo (CCHN–UFES), Av. Fernando Ferrari, 514, Goiabeiras, Vitória, Espírito Santo 29075-900 Brazil; 5 Marine Fish Ecology Laboratory (LEPMAR), Department of Agricultural and Biological Sciences, Federal University of Espírito Santo, BR-101, Km 60, Litorâneo, São Mateus, Espírito Santo 29932-540 Brazil; 6 Laboratory of Ecotoxicology and Applied Ecology (LEEA), Department of Hydraulics and Sanitation, São Carlos School of Engineering, University of São Paulo, São Carlos, SP Brazil; 7 Graduate Program in Environmental Sciences and Technology, University of Vale do Itajaí (UNIVALI), Itajaí, SC Brazil

**Keywords:** Brazil, Beach, Crustacean, Biological indicators, Human pressures

## Abstract

Sandy beach ecosystems are increasingly affected by climatic variability and human pressures. The ghost crab *Ocypode quadrata* is widely used as a bioindicator of environmental conditions on sandy beaches. However, important gaps remain in understanding how natural sources of variability, such as seasonality and beach morphodynamics, influence its population dynamics. We evaluated seasonal and spatial variation in burrow density, biomass, carapace dimensions, and body condition, used here as morphological and population descriptors of *O. quadrata*, along morphodynamic gradient of sandy beaches in southern Brazil. Biomass and carapace dimensions, obtained through direct census, varied significantly among beaches and seasons, with higher values generally recorded on the reflective beach during summer, whereas intermediate and dissipative beaches showed higher values during winter. In contrast, body condition consistently peaked in autumn across all beaches. Burrow density, estimated through indirect census, did not vary significantly across seasons or beaches, but was positively associated with vegetation area, as were the other descriptors examined. These findings suggest that seasonal dynamics and habitat characteristics, including vegetation area, may be associated with variation in *O. quadrata* population descriptors across beaches differing in morphodynamic condition. Accounting for these natural sources of variability may support more reliable use of *O. quadrata* as a bioindicator in sandy beach ecosystems.

**Supplementary Information:**

The online version contains supplementary material available at https://doi.org/10.1007/s10661-026-15876-z.

## Introduction

Coastal and estuarine environments are among the most vulnerable ecosystems worldwide because they are increasingly exposed to multiple and interacting human pressures (Ostrowski et al., 2021; Worm et al., 2006). Sandy beaches and dune systems are particularly affected, as their position at the land–sea interface directly links natural dynamics with human activities (Defeo & Elliott, 2021; McLachlan & Defeo, 2018). Habitat loss driven by sand compaction, off-road vehicle (ORV) traffic, recreational activities (Defeo et al., 2009), removal of native vegetation (Orlando et al., 2020), and coastal armouring through hard defence structures has reduced environmental quality and species diversity within these ecosystems (Laurino et al., 2022; Wangkulangkul et al., 2025). In addition, climate-related changes in temperature and precipitation intensify erosion and accelerate beach degradation (Fanini et al., 2020).

Although not all sandy beaches are poorly managed, many have undergone continuous transformation due to what has been described as the “triple whammy”: increasing urbanization and industrialization, excessive exploitation of natural resources, and declining resilience to climate change (Defeo & Elliott, 2021; McLachlan & Defeo, 2018). These pressures are often exacerbated by gaps in beach management, as sandy beaches are frequently incorporated into broader coastal frameworks without explicit consideration of their ecological dynamics. This combination of anthropogenic stressors and management challenges highlights the need to recognize the unique characteristics of sandy beaches (Fanini et al., 2020). Among the tools available, robust ecological metrics, particularly biological indicators, are essential for assessing beach condition and supporting effective conservation strategies (Schlacher et al., 2014). The responses of sandy beach organisms to environmental variability may involve phenotypic plasticity, through which individuals can adjust morphological, physiological, or behavioural characteristics under changing habitat conditions (Scapini et al., 2019). However, observed variation in biological responses may also reflect differences in habitat quality, resource availability, and population processes.

Biological responses of fauna and flora have long been used to assess anthropogenic and climatic impacts (Dale & Beyeler, 2001; Niemi & McDonald, 2004), as they integrate environmental stress over time and reflect the magnitude of ecological disturbance (Lindenmayer, 1999; Siddig et al., 2016). In sandy beach and dune systems, crustaceans have emerged as especially valuable indicators due to their abundance, ecological relevance, and sensitivity to human activities (Ocaña et al., 2020; Suciu et al., 2018). In particular, ghost crabs of the genus *Ocypode* are widely used as ecological indicators on tropical and subtropical sandy beaches (Lucrezi & Schlacher, 2014), as their broad distribution, sensitivity to environmental change, and easily measurable morphological and population descriptors including body size and burrow density make them useful indicators across beaches and dunes (de Souza et al., 2017; Jonah et al., 2015).

Ghost crabs are also recognized as umbrella species on sandy beaches (Costa & Zalmon, 2021), such that their conservation may contribute to the protection of a broader set of organisms inhabiting these environments (Fleishman et al., 2001; Simberloff, 1998). Consistent with this role, they respond negatively to a range of anthropogenic stressors, including trampling (Lucrezi et al., 2009), vehicular traffic (Costa et al., 2020), and the suppression of coastal vegetation (Barboza et al., 2021). Beyond their value as bioindicators, ghost crabs play a fundamental ecological role by linking marine and terrestrial food webs as omnivorous predators and scavengers (do Vale et al., 2022). Vegetation area further enhances these functions by stabilizing burrows, providing shelter (Lucrezi et al., 2010; Schlacher et al., 2011), and supporting trophic interactions among macrofauna, birds, and other organisms associated with sandy beaches (Gül & Griffen, 2019; Orlando et al., 2020).

The increasing use of ghost crabs as indicators of human disturbance (Barboza et al., 2021) and extreme climatic events reflects the feasibility of assessing their populations using non-invasive and cost-effective methods (Machado et al., 2019). Indirect census approaches based on burrow counts and burrow entrance diameter provide reliable estimates of abundance and population structure and are widely applied in environmental monitoring (Lucrezi et al., 2009; Schlacher et al., 2016). These approaches are complemented by direct census methods based on individual capture, which allow the assessment of biomass, body dimensions, and individual condition, commonly quantified using the relative condition factor (Kn) (Gül & Griffen, 2020) and responsive to environmental variability and anthropogenic stress (Barrilli et al., 2015). Although these descriptors are biologically related, they provide complementary information on different aspects of ghost crab responses to environmental variability. Burrow density reflects population-level responses associated with habitat occupancy and dynamics (da Rosa & Borzone, 2008; Lucrezi et al., 2009), whereas body size and biomass provide information on population structure and individual development, integrating the effects of environmental conditions over time (Oliveira et al., 2003; Schirf et al., 1987). Relative condition factor provides an estimate of the relative condition of individuals, which may be influenced by factors such as food availability, reproduction, and moulting (Gül & Griffen, 2020; Hamid et al., 2018). Despite their widespread use, uncertainties remain regarding how natural sources of variation, such as seasonal dynamics and morphodynamic differences among beaches, influence the morphological and population descriptors of ghost crabs. Such environmental factors may influence burrow density, body size, biomass (de Oliveira et al., 2016), and individual condition (Costa et al., 2022) independently of human disturbance, potentially confounding the interpretation of biological responses to anthropogenic impacts (Checon et al., 2023). Furthermore, most studies evaluate these factors in isolation, and few have examined how multiple morphological and population descriptors respond simultaneously across beaches with contrasting morphodynamic characteristics. The role of habitat features such as coastal vegetation, which can influence sediment stability and burrow persistence, also remains insufficiently explored in this context. Addressing these gaps is essential to refine the application of ghost crabs as bioindicators and to better understand the ecological patterns on sandy beaches.

Thus, this study aims to investigate seasonal variation in the morphological and population descriptors of the ghost crab *Ocypode quadrata* across sandy beaches spanning a morphodynamic gradient in southern Brazil. We evaluate how morphological and population descriptors of *O. quadrata* reflect environmental conditions and examine how these descriptors respond to local habitat features, with particular emphasis on beach vegetation. We hypothesize that vegetation area significantly influences the morphological and population descriptors of *O. quadrata*.

## Materials and methods

### Study area

**Fig. 1:**
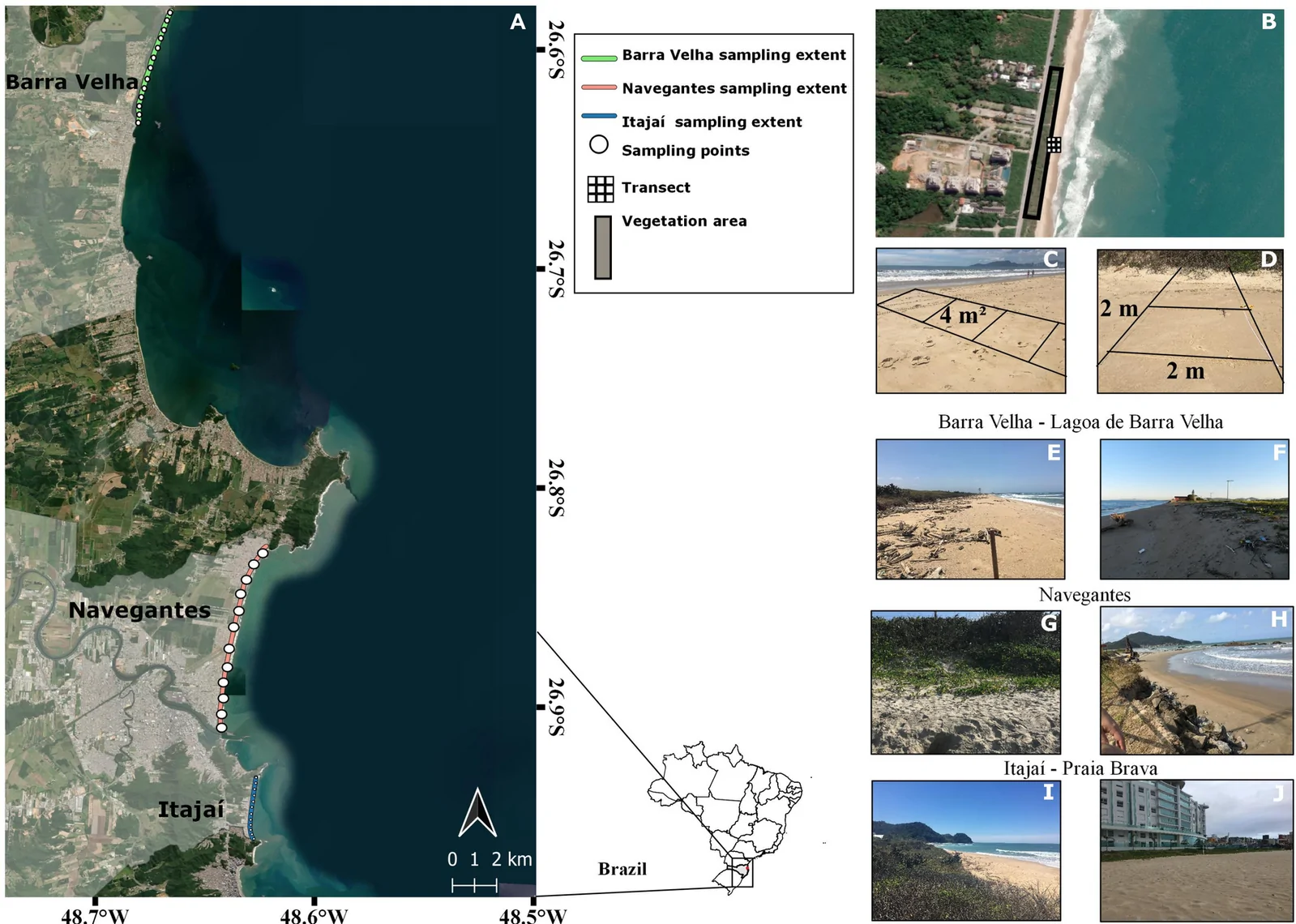
Study area and sampling design. **A** Location of the three sandy beaches surveyed on the central–northern coast of Santa Catarina, southern Brazil. Coloured lines indicate the survey extent at each beach, and circles represent the sampling sites. **B** Example of the sampling design showing the vegetation area and the 2 × 2-m transect used for burrow density estimates. **C**–**D** Field photographs illustrating the 4-m^2^ transect. **E**–**J** Representative views of Barra Velha, Navegantes, and Brava Beach (Itajaí). Photo credits: Bing Maps Aerial (**A**, **B**); photographs by JGV (**C**–**J**)

Monthly sampling was conducted from March 2017 to February 2018 on three sandy beaches along the central–northern coast of Santa Catarina, southern Brazil (Fig. 1A), under license number 57786 issued by the Biodiversity Authorization and Information System (SISBIO). The beaches were previously classified along a morphodynamic gradient as reflective (Barra Velha), dissipative (Navegantes), and intermediate (Brava Beach, Itajaí) based on sediment grain size, beach slope, and the width of the sandy shore (do Vale et al., 2022).

Barra Velha Beach, located in the municipality of Barra Velha (26°34′56.64″–26°37′37.70″ S; 48°39′55.38″–48°40′49.12″ W), is approximately 6.0 km long and characterized by a steep slope, coarse sand grains, a narrow surf zone, and dunes covered by shrubs and medium-sized trees (Fig. 1B–C). The beach is primarily used by local residents and fishermen. Navegantes Beach (26°49′36.5″–26°54′35.46″ S; 48°37′17.36″–48°38′34.61″ W) extends for 10.0 km and presents fine sand grains, a gentle slope, and extensive dunes with ground vegetation, shrubs, and medium-sized trees (Fig. 1D–E). It is intensively used for recreational and tourism activities. Brava Beach, located in the municipality of Itajaí (26°55′57.30″–26°57′36.02″ S; 48°37′35.08″–48°37′41.35″ W), is approximately 3.0 km long and exhibits a moderate slope, medium sand grains, and a moderate surf zone. Dunes are mainly covered by ground vegetation and shrubs, and the beach attracts a high number of bathers and tourists due to its infrastructure (Fig. 1F–G). Navegantes Beach and Brava Beach (Itajaí) are separated by the Itajaí-Açu estuarine system, one of the main estuarine environments in southern Brazil. This system is characterized by strong interactions between river discharge and marine processes, generating environmental gradients in salinity, water properties, and material transport (Pereira Filho & Rörig, 2016). The estuary also contributes to the input and redistribution of nutrients and organic matter in adjacent coastal environments, potentially influencing local habitat conditions.

### Sampling procedures

#### Vegetation area

Each beach was divided into three alongshore sections proportional to its total shoreline extension, with sections of approximately 1 km in Itajaí, 2 km in Barra Velha, and 3.3 km in Navegantes. These sections were established to distribute sampling effort along the beach extension. Because beaches differed substantially in shoreline extension, standardizing sampling by a fixed distance was not logistically feasible; instead, sampling effort was standardized by survey duration. At each monthly sampling event, one section was surveyed, in a rotating sequence across the three sections, by the same two researchers over a 2-h period, coinciding with periods of peak surface activity of *O. quadrata*. This resulted in four sampling events per section and 12 surveys per beach over the 1-year study period.

Vegetation area (m^2^) was calculated as the product of the alongshore distance covered during sampling (length) and the vegetation width measured perpendicular to the shoreline (width), thus representing a rectangular approximation of the vegetated area within the sampled stretch. Because survey effort was standardized by duration rather than by a fixed distance, the alongshore distance covered by the survey team was comparable across surveys, so that variation in vegetation area primarily reflected differences in vegetation width among beaches rather than differences in the extent of the sampled stretch.

#### Morphological and population descriptors

To minimize potential biases associated with indirect (burrow counting) and direct (specimen sampling) censuses, sampling was conducted during periods of peak surface activity of ghost crabs, including burrow construction, maintenance, and foraging (Evans et al., 1976; Jones, 1972; Lucrezi & Schlacher, 2014). Surface activity occurs predominantly during the daytime in colder seasons (autumn–winter) and at night in warmer seasons (spring–summer) (do Vale et al., 2022), and sampling periods were adjusted accordingly.

Within each 2-h survey, burrow density was first estimated at a randomly selected location within the section, using three replicate transects (2 × 2 m) established following the cross-shore distribution of burrows, from the waterline towards the supralittoral and dune zones, following a protocol adapted from Pombo and Turra (2013). Burrow density was expressed as the mean number of burrows per square meter. Active crab collection was then conducted over the remainder of the 2-h period by the same two researchers, by manually capturing individuals moving across the beach surface or excavating burrows showing signs of recent activity (do Vale et al., 2022), covering a larger alongshore extent of the section than the burrow transects. Captured crabs were individually placed in labelled plastic bags and transported to the laboratory in a thermal box with ice to induce hypothermia and reduce metabolic activity, following chilling procedures described for decapod crustaceans (EFSA AHAW Panel, 2005). Specimens were subsequently stored in a freezer (− 20 °C) until biometric analyses. In the laboratory, carapace width (CW) and carapace length (CL) were measured using a digital calliper with 0.01 mm precision. Biomass (fresh body weight) was determined using a high-precision analytical balance. Finally, the relative condition factor (Kn) was used to assess the physiological condition of *O. quadrata*. The weight–carapace width relationship was described by the equation $W=a\cdot C{W}^{b}$ (Le Cren, 1951), where coefficients *a* and *b* were used to estimate expected weight (We) from CW. Kn was calculated as the ratio between observed weight (Wo) and expected weight (We) for each individual.

### Data analysis

Differences in vegetation area and burrow density across sandy beaches characterized by contrasting morphodynamic conditions and among seasons (spring, summer, autumn, and winter) were evaluated using permutational multivariate analysis of variance (PERMANOVA; Anderson, 2001). In addition, biomass (W_o_), carapace length (CL), carapace width (CW), and relative condition factor (Kn) were evaluated using separate, independent PERMANOVA models. Prior to analysis, the distributional properties of the response variables were evaluated, revealing departures from normality and homogeneity of variances among groups. PERMANOVA was selected because, as a permutation-based method, it does not depend on the assumption of a specific error distribution and remains robust under such departures, allowing beach, season, and their interaction to be tested within the same balanced factorial design without requiring data transformation.

All PERMANOVA analyses were performed using Euclidean distance matrices and 9.999 permutations with the *adonis2* function of the vegan package (Oksanen et al., 2020) in R version 4.3.1 (R Core Team, 2023). Beach (Barra Velha, Navegantes, and Itajaí), season (spring, summer, autumn, and winter), and their interaction were included as fixed factors in the models.

To evaluate the influence of vegetation area on ghost crab morphological and population descriptors, separate generalized linear models (GLMs) were fitted each descriptor as an individual response variable, assuming a Gaussian error distribution appropriate for continuous data. Monthly mean values were used as sampling units. Vegetation area was included as a continuous predictor, and beach identity was included as a categorical factor to account for spatial differences among study sites. Because vegetation area varied continuously among sampling months, model predictions represent responses along a vegetation area gradient rather than temporal trends.

## Results

### Vegetation area

Vegetation area varied markedly among beaches and across months. Barra Velha showed values ranging from 35.13 to 591.33 m^2^ (mean ± SD: 182.40 ± 164.84), with higher values in winter and lower values in spring. In Navegantes, vegetation area ranged from 32.22 to 539.74 m^2^ (296.87 ± 161.18), with the highest values recorded in summer and the lowest in winter. Itajaí exhibited lower overall vegetation cover, ranging from 37.64 to 245.59 m^2^ (128.15 ± 65.10), with maximum values in summer and minimum values in autumn. PERMANOVA indicated significant differences in vegetation area among beaches (*F* = 3.415; *p* = 0.028), whereas seasonal variation was not significant (Table 1). Pairwise comparisons showed that Navegantes had significantly higher vegetation area than both Barra Velha (*F* = 3.266; *p* < 0.01) and Itajaí (*F* = 7.211; *p* < 0.01).

**Table 1:** Results of permutation multivariate analysis of variance among beaches (Barra Velha, Navegantes, and Itajaí) and sampling seasons (spring, summer, autumn, and winter), based on Euclidian distance matrix for vegetation area and burrows per square meter. Caption: *df*, degrees of freedom; *SS*, sum of squares. Significant *p*-values in bold

Source	df	Vegetation area/m^2^			Burrows/m^2^		
		Sum Of Sqs	F	*p*	Sum Of Sqs	F	*p*
Season (S)	3	0.221	0.759	0.575	0.038	0.308	0.868
Beach (B)	2	0.664	3.415	**0.028**	0.163	2.002	0.141
Interaction (SxB)	6	0.586	1.006	0.451	0.097	0.398	0.908
Residual	24	2.332			0.976		
Total	35	3.803			1.274		

### Ghost crab morphological and population descriptors

Burrow density showed seasonal and spatial variation but no significant effects of season, beach, or their interaction according to PERMANOVA (Supplementary Table S1). Mean burrow densities ranged from 0.13 to 0.50 burrows m^2^ across beaches and seasons (Supplementary Table S2).

**Fig. 2:**
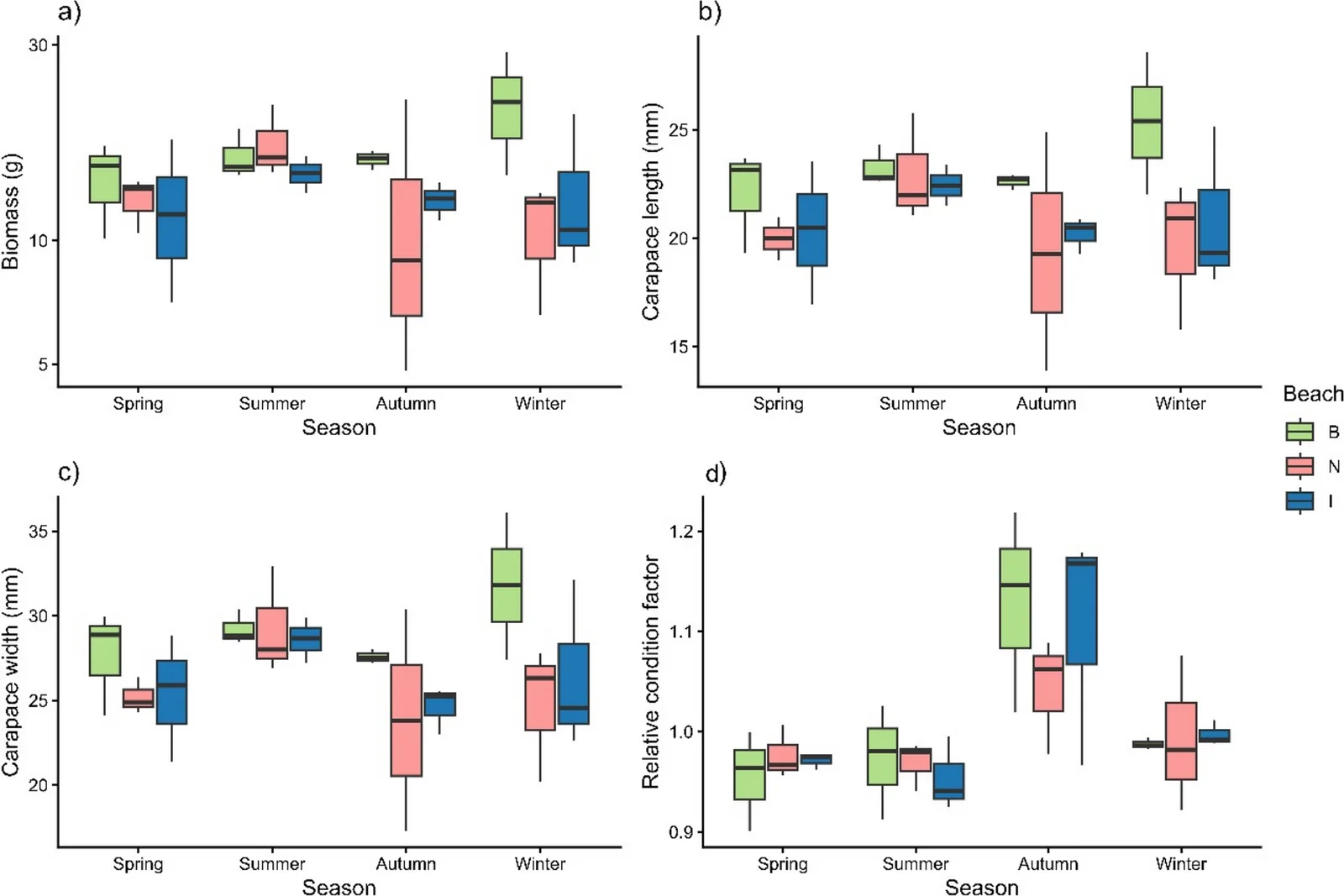
Boxplots showing the distribution of morphological and population descriptors of *Ocypode quadrata*: biomass (**a**), carapace length (**b**), carapace width (**c**), and relative condition factor (**d**) across seasons on the beaches of Barra Velha (B), Navegantes (N), and Itajaí (I), southern Brazil

A total of 660 individuals were captured for direct morphological analyses (Supplementary Table S1). Correlation analyses based on monthly mean values revealed significant positive relationships among biomass (Wo), carapace length (CL), carapace width (CW), and relative condition factor (Kn) (Supplementary Fig. S1). These descriptors are therefore interpreted as complementary descriptors of body size and body condition rather than as independent lines of evidence (Figure S1; supplementary X). Biomass, carapace length, carapace width, and relative condition factor (Kn) varied significantly across seasons and/or beaches (Table 2; Fig. 2).

**Table 2:** Results of permutation multivariate analysis of variance among beaches (Barra Velha, Navegantes, and Itajaí) and sampling seasons (spring, summer, autumn and winter), based on Euclidian distance matrix for biomass, carapace length, carapace width, condition factor considering the width (Kn) of *Ocypode quadrata* individuals. Caption: *df*, degrees of freedom; *SS*, sum of squares. Significant *p*-values in bold

Source	df	Biomass			Length			Width			Kn		
		SS	Pseudo-F	*p*	SS	Pseudo-F	*p*	SS	Pseudo-F	p(perm)	SS	Pseudo-F	*p*
Season (S)	3	2997.3	5.0934	**0.002**	3709.6	4.8049	**0.001**	5111.9	6.7625	**0.001**	4514.9	33.411	**0.001**
Beach (B)	2	4813.9	10.827	**0.004**	6631.4	10.169	**0.002**	6164.4	8.8706	**0.006**	88.293	0.53433	0.66
(S) x (B)	6	1343	1.1411	0.319	1980.2	1.2824	0.226	2118	1.4009	0.177	508.79	1.8826	0.073
Residual	648	127,110			166,760			163,280			29,188		
Total	659	135,450			178,040			175,810			35,112

Biomass, carapace length, and carapace width exhibited similar spatial and seasonal responses (Table 2; Fig. 2a–c). All three descriptors differed significantly among beaches and seasons, whereas their detailed temporal patterns are presented in Fig. 2.

The relative condition factor (Kn) was significantly affected by season only (*F* = 33.411; *p* = 0.001). Pairwise comparisons indicated that Kn values in autumn were significantly higher than in winter, spring, and summer and that summer also differed from winter. Across all beaches, the highest Kn values were consistently observed during autumn (Fig. 2d)*.*

### Relationships between vegetation and ghost crab descriptors

Generalized linear models indicated positive relationships between vegetation area and the morphological and population descriptors of *O. quadrata* (Table 3).

**Table 3:** Results from generalized linear models (GLM) testing the relationships among biomass measurements, carapace length, carapace width, average burrows per square meter, and relative condition factor with the vegetation area of the sampled beaches. Significant *p*-values in bold

**Burrow density**				
	Estimate	Std. Error	*t* value	Pr(>|t|)
(Intercept)	− 1.3700	0.1402	− 9.7740	** < 0.01**
Vegetation (V)	0.0010	0.0005	2.0510	**0.04**
Itajaí (I)	− 0.3662	0.2901	− 1.2630	0.22
Navegantes (N)	− 0.4682	0.2793	− 1.6760	0.10
Biomass				
	Estimate	Std. Error	*t* value	Pr(>|t|)
(Intercept)	2.5513	0.0438	58.2600	** < 0.01**
Vegetation (V)	0.0014	0.0002	9.0060	** < 0.01**
Itajaí (I)	− 0.5347	0.0892	− 5.9930	** < 0.01**
Navegantes (N)	− 0.7526	0.0919	− 8.1870	** < 0.01**
Carapace length				
	Estimate	Std. Error	*t* value	Pr(>|t|)
(Intercept)	3.0550	0.0242	26.4460	** < 0.01**
Vegetation (V)	0.0005	0.0001	5.3240	** < 0.01**
Itajaí (I)	− 0.2344	0.0458	− 5.1210	** < 0.01**
Navegantes (N)	− 0.3101	0.0454	− 6.8310	** < 0.01**
Carapace width				
	Estimate	Std. Error	*t* value	Pr(>|t|)
(Intercept)	3.2700	0.0254	28.9870	** < 0.01**
Vegetation (V)	0.0005	0.0001	5.3090	** < 0.01**
Itajaí (I)	− 0.2347	0.0480	− 4.8920	** < 0.01**
Navegantes (N)	− 0.2979	0.0475	− 6.2740	** < 0.01**
Relative condition factor				
	Estimate	Std. Error	*t* value	Pr(>|t|)
(Intercept)	− 0.0887	0.0240	− 3.6960	** < 0.01**
Vegetation (V)	0.0005	0.0001	5.3080	** < 0.01**
Itajaí (I)	0.0037	0.0432	0.0860	0.932
Navegantes (N)	0.0240	0.0419	0.5730	0.571

**Fig. 3:**
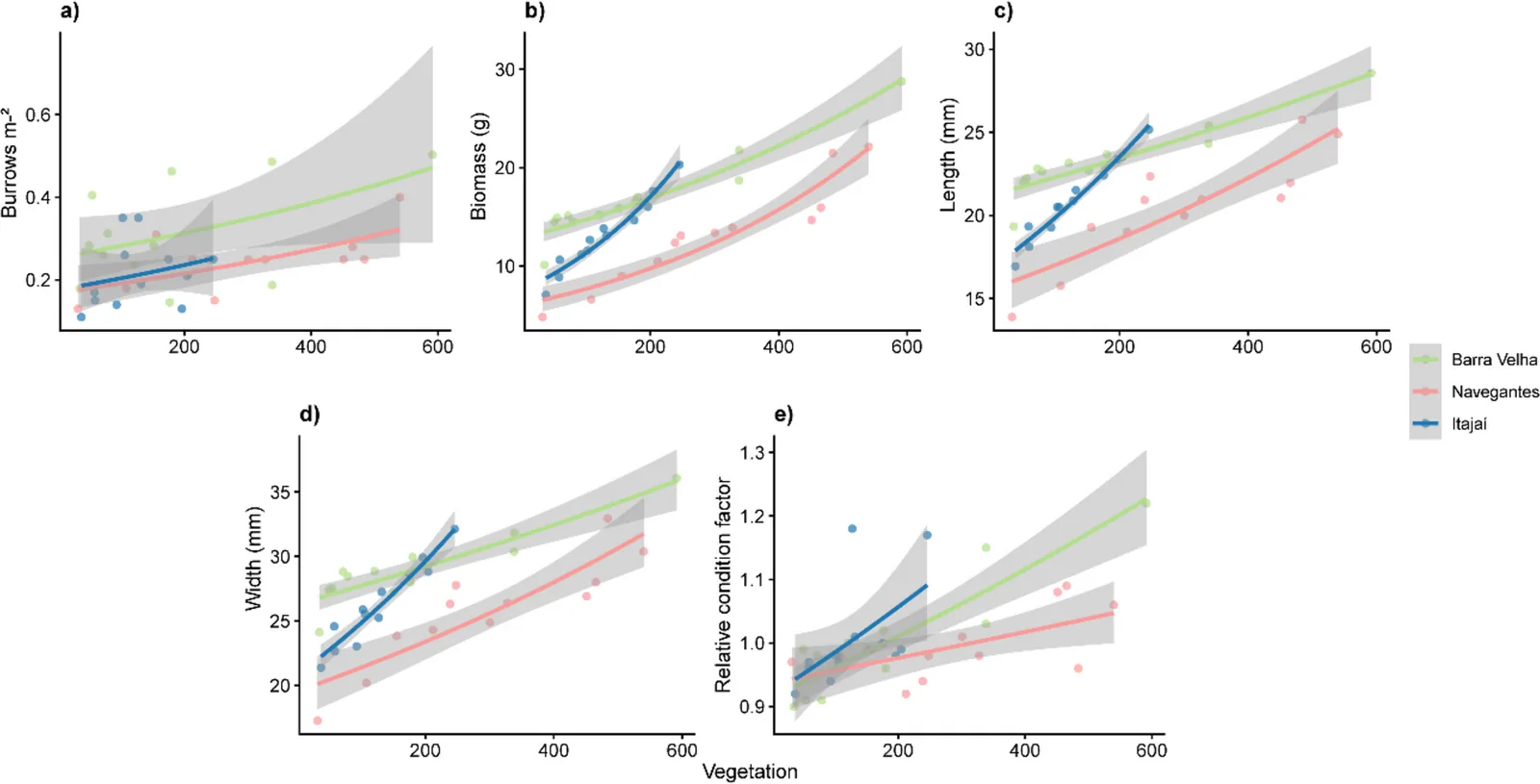
Generalized linear model relationships between measurements of biological traits and the vegetation area on sandy beaches. Legend: burrow density (**a**), biomass (**b**), carapace length (**c**), carapace width (**d**), and relative condition factor, and (**e**) of *Ocypode quadrata* on beaches

Burrow density increased significantly with increasing vegetation area (*p* = 0.04; Fig. 3a). Biomass, carapace length, and carapace width were also positively related to vegetation area across the three beaches, with significant increases observed along the vegetation area gradient (*p* < 0.01; Fig. 3b–d).

Relative condition factor (Kn) also increased significantly with vegetation area (Fig. 3). Overall, GLM results indicated morphological and population descriptors of *O. quadrata* increased with vegetation area across the sampled beaches.

## Discussion

The present study demonstrates that morphological and population descriptors of *O. quadrata* responded to natural spatial and seasonal variation across the study beaches. Biomass, carapace length, and carapace width exhibited consistent spatial and seasonal patterns and were positively associated with vegetation area, whereas the relative condition factor (Kn) varied only among seasons. Although these descriptors were significantly correlated, they differed in their ability to reflect environmental variation, with size-related descriptors capturing both spatial and seasonal patterns, while Kn detected only seasonal variation. Together, these findings highlight the importance of integrating multiple biological descriptors to better understand how *O. quadrata* populations respond to habitat characteristics and temporal variability.

Burrow density showed differences in mean values among seasons, with higher values observed during autumn and summer, although these differences were not statistically significant. Similar seasonal patterns have been documented in previous studies (da Rosa & Borzone, 2008; de Oliveira et al., 2016), where increases in burrow densities have been associated with processes such as juvenile (Branco et al., 2010). In our study, sampling was conducted during periods of higher surface activity of ghost crabs in southern Brazil, with nocturnal sampling in spring–summer and diurnal sampling in autumn–winter (do Vale et al., 2022). This sampling strategy was designed to reduce potential differences in surface activity and detectability among seasons, increasing confidence that observed patterns reflect population dynamics rather than methodological inconsistencies. Therefore, burrow density remains a useful monitoring metric when seasonal variability is considered in ecological assessments.

In contrast, variation among beaches was more evident. The reflective beach supported higher burrow densities than the intermediate and dissipative beaches. Similar patterns were reported by Turra et al. (2005) and da Rosa and Borzone (2008), although contrasting patterns have also been documented (Lucrezi, 2015; Pombo et al., 2017). These contrasting results suggest that the relationship between beach morphodynamics and *Ocypode* burrow density may vary according to local environmental conditions. Such patterns are consistent with environmental filtering, in which morphodynamic characteristics influence habitat suitability for supralittoral species (Defeo & Gómez, 2005; Defeo & McLachlan, 2011; McLachlan et al., 1993). Additionally, spatial differences between Navegantes and Itajaí may also be influenced by the presence of the Itajaí-Açu estuary, which separates these beaches. Estuarine influence can modify adjacent sandy beach environments through changes in sediment dynamics, freshwater inputs, and resource availability (Pereira Filho & Rörig, 2016). Although these effects were not quantified here, they represent a potential source of environmental variability that should be considered in future studies.

Biomass, carapace length and width exhibited clear seasonal patterns across the studied beaches. Growth in ghost crabs reflects the balance between energy acquisition and allocation to maintenance, growth, moulting, and reproduction and is therefore influenced by both environmental conditions and endogenous processes (Kucharski & Da Silva, 1991; Oliveira et al., 2003; Schirf et al., 1987). In the present study, the highest biomass, carapace length, and carapace width values occurred during winter and summer, coinciding with increased consumption of crustaceans reported for the same beaches (do Vale et al., 2022). Because animal prey provides greater energetic returns than plant material (Arueira et al., 2022; Rae et al., 2019; Wolcott, 1978), this temporal correspondence suggests that seasonal variation in diet may contribute to observed growth patterns in *O. quadrata*, consistent with previous observations (Gül & Griffen, 2020). Beyond environmental conditions, biotic interactions such as predator–prey dynamics may also contribute to shaping sandy beach macrofauna populations (Arueira et al., 2025). These findings highlight that natural seasonal variation should be considered when interpreting body size descriptors, particularly when they are used to assess environmental condition in sandy beach ecosystems.

Individuals from the reflective beach (Barra Velha) showed larger carapace dimensions and higher biomass than those from intermediate and dissipative beaches, despite lower stomach fullness reported for this site (do Vale et al., 2022). This apparent contradiction is consistent with the habitat safety hypothesis (HSH) (Defeo & Gómez, 2005), which proposes that individuals in physically stable environments allocate energy preferentially to growth rather than maintenance or disturbance avoidance (Defeo & McLachlan, 2011). Thus, larger body size at Barra Velha may reflect the outcome of habitat filtering rather than short-term feeding intensity, suggesting that morphodynamic context may contribute to variation in energy allocation strategies in *O. quadrata*.

In contrast to biomass and carapace dimensions, Kn did not vary among beaches but showed significant seasonal variation, indicating that these descriptors capture different components of *O. quadrata* variability. While biomass and carapace dimensions reflected spatial and seasonal variation in body size, Kn primarily reflected seasonal changes in individual condition. Seasonal variation in body condition (Kn) provides additional insight into potential energetic trade-offs in ghost crabs. Seasonal fluctuations in body condition are commonly associated with changes in environmental conditions, food availability and foraging behaviour, and endogenous processes such as reproduction and moulting (Hamid et al., 2018). Contrary to expectations of higher body condition during spring–summer, Kn values were lowest during this period. *O. quadrata* reproduces primarily during spring–summer (Branco et al., 2010), and both reproduction and moulting involve substantial energetic demands that may reduce somatic reserves (Hamid et al., 2018; Pinheiro & Fiscarelli, 2009; Pinheiro et al., 2005). Therefore, the seasonal decrease in Kn may be associated with energy allocation towards reproduction and somatic maintenance rather than solely reduced resource availability. These results highlight that body size descriptors and Kn provide complementary information on population variability, as they represent different aspects of *O. quadrata* responses.

Taken together, these patterns indicate that the descriptors examined differ in their sensitivity to environmental variation, which has direct implications for their use in monitoring. Burrow density showed no significant variation across either seasons or beaches, suggesting low natural background variability and supporting its potential reliability for detecting deviations associated with anthropogenic disturbance, although this pattern should be interpreted with caution given the sampling design. In contrast, biomass and carapace dimensions were sensitive to both spatial and seasonal habitat conditions, indicating that comparisons involving these descriptors require explicit control for season and beach type to avoid confounding natural variability with disturbance effects. Kn varied only seasonally and was stable among beaches, making it comparatively more reliable for spatial comparisons but requiring seasonal control when used to track temporal change. Accounting for these descriptor-specific sensitivities is therefore essential for the appropriate use of *O. quadrata* as a bioindicator, as failing to do so may lead to biased interpretations of environmental change, particularly when comparisons involve different seasons or beach characteristics. Using these descriptors in combination, rather than in isolation, may thus provide a more robust basis for interpreting *O. quadrata* responses while minimizing the risk of confounding natural variability with anthropogenic effects.

Vegetation area was also associated with variation in morphological and population descriptors of *O. quadrata*. This finding aligns with previous studies highlighting the importance of coastal vegetation in providing suitable habitat conditions for ghost crabs (Barboza et al., 2021; Schlacher et al., 2011). Vegetation may enhance habitat stability by reducing sediment mobility and erosion, potentially facilitating burrow persistence and reducing energetic costs associated with burrow maintenance. As an umbrella species, *O. quadrata* connects marine and terrestrial food webs and plays a fundamental ecological role in sandy beach ecosystems (Costa & Zalmon, 2021; do Vale et al., 2022).

Environmental degradation of sandy beaches, driven by urban development and climate change, has intensified processes such as coastal squeeze, leading to dune reconfiguration and vegetation loss (Defeo & Elliott, 2021). Because dune vegetation is an important component of supralittoral habitat conditions, its loss may affect habitat suitability for ghost crabs and associated biodiversity (de Souza et al., 2017; Schlacher et al., 2011). By demonstrating associations between vegetation area and morphological and population descriptors of *O. quadrata*, our results reinforce the link between habitat characteristics and ghost crab responses, while highlighting that assessments should incorporate natural spatial and seasonal variability. Accordingly, preserving beach and dune vegetation, and incorporating vegetation cover as a practical criterion in coastal monitoring and restoration programmes, is essential for maintaining habitat conditions that support ghost crab populations and associated biodiversity.

## Conclusion

Our study indicates that responses of *O. quadrata* vary according to seasonal and local habitat characteristics, highlighting the importance of accounting for natural variability when interpreting this species in environmental assessments. Burrow density showed no significant variation across seasons or beaches, indicating comparatively low natural background variability, which may reduce the risk of confounding natural fluctuations with other sources of environmental change. Biomass and carapace dimensions responded to both seasonal and spatial habitat conditions and therefore required explicit control for these factors when used in environmental comparisons and body condition (Kn) varied only seasonally and was stable among beaches, making it comparatively more reliable for spatial comparisons but requiring seasonal control when tracking temporal change. The positive associations between vegetation area and morphological and population descriptors further emphasize the ecological importance of beach–dune vegetation for maintaining suitable habitat conditions for ghost crabs, supporting consideration of vegetation cover as a relevant factor in coastal monitoring efforts. Together, these findings suggest that a combination of descriptors, rather than any single metric in isolation, provides a more robust and comprehensive basis for interpreting *O. quadrata* responses while accounting for natural temporal and spatial variability. This approach may improve the reliability of *O. quadrata* as a bioindicator in environmental assessments of sandy beach ecosystems.

## Supplementary Information

Below is the link to the electronic supplementary material.

ESM 1 (DOCX 189 KB)

## Data Availability

The authors confirm that the data supporting the findings of this study are available within the article [and/or] its supplementary materials. Raw reads are available upon reasonable requests from the corresponding authors.
