# Supplementary material for: Seasonal, morphodynamic, and vegetation drivers of ghost crab (Ocypode quadrata) populations on subtropical sandy beaches
Source: Environ Monit Assess. 2026 Sep 4;198(9):1038. doi: 10.1007/s10661-026-15876-z (PMC13545024; doi:10.1007/s10661-026-15876-z)
Supplement: Supplementary file 1 [file 10661_2026_15876_MOESM1_ESM.docx]

**Supplementary data**

**Table S1** Data of *Ocypode quadrata* captured along March 2017 to February 2018 on the beaches of Barra Velha, Navegantes and Itajaí. Amplitude, mean (x̄), standard error of biomass, length, carapace width and relative condition factor

| Season | Beach | Sample size | Biomass (g) | | Carapace Length (mm) | | Carapace Width (mm) | | Relative Condition Factor | |
| --- | --- | --- | --- | --- | --- | --- | --- | --- | --- | --- |
|  |  | n | Min-Max | x̄ | Min-Max | x̄ | Min-Max | x̄ | Min-Max | x̄ |
| Spring | Barra Velha | 49 | 0.86-42.13 | 14.56±1.42 | 10.03-33.75 | 22.30±0.77 | 12.30-41.37 | 28.00±0.95 | 0.69-1.20 | 0.96±0.01 |
|  | Navegantes | 39 | 0.39-44.90 | 12.58±1.87 | 7.66-35.59 | 19.98±1.20 | 9.46-42.86 | 25.19±1.53 | 0.55-1.24 | 0.97±0.02 |
|  | Itajaí | 47 | 0.25-37.70 | 11.53±1.48 | 6.19-35.40 | 19.98±0.99 | 7.85-39.42 | 24.97±1.16 | 0.73-1.14 | 0.97±0.01 |
| Summer | Barra Velha | 62 | 2.69-44.44 | 14.77±1.53 | 15.61-36.09 | 22.72±0.79 | 18.38-42.26 | 28.62±1.01 | 0.66-1.24 | 0.94±0.01 |
|  | Navegantes | 76 | 0.15-49.78 | 17.03±1.46 | 3.63-34.94 | 22.70±0.78 | 5.87-45.01 | 29.00±0.99 | 0.73-1.53 | 0.97±0.01 |
|  | Itajaí | 96 | 0.13-45.26 | 14.47±0.85 | 5.67-33.56 | 22.36±0.46 | 6.73-43.22 | 28.49±0.61 | 0.23-1.14 | 0.95±0.01 |
| Autumn | Barra Velha | 63 | 0.92-43.76 | 15.52±1.33 | 10.48-32.95 | 22.51±0.65 | 13.17-40.87 | 27.48±0.78 | 0.75-1.61 | 1.12±0.02 |
|  | Navegantes | 44 | 0.08-45.64 | 14.11±1.95 | 4.83-34.72 | 20.48±1.21 | 5.61-43.04 | 25.17±1.45 | 0.74-1.37 | 1.05±0.02 |
|  | Itajaí | 73 | 0.10-46.53 | 12.48±1.11 | 4.79-33.69 | 20.10±0.77 | 5.90-39.40 | 24.32±0.90 | 0.31-1.57 | 1.14±0.02 |
| Winter | Barra Velha | 47 | 0.44-45.96 | 21.21±1.81 | 7.27-34.41 | 25.12 ±0.89 | 9.43-42.80 | 31.50±1.12 | 0.72-1.10 | 0.98±0.01 |
|  | Navegantes | 27 | 0.29-33.36 | 10.24±1.42 | 7.04-30.53 | 19.24±1.11 | 8.38-37.92 | 24.26±1.43 | 0.65-1.37 | 0.99±0.02 |
|  | Itajaí | 37 | 0.37-40.00 | 12.51±1.69 | 7.23-32.50 | 20.15±1.07 | 8.76-41.50 | 25.47±1.38 | 0.81-1.23 | 1.00±0.01 |
| Total | Barra Velha | 221 | 0.44-45.96 | 16.77±0.72 | 7.27-36.09 | 23.26±0.36 | 9.43-42.80 | 28.97±0.45 | 0.66-1.61 | 1.01±0.00 |
|  | Navegantes | 186 | 0.08-49.78 | 14.42±0.88 | 3.63-35.59 | 21.10±0.52 | 5.61-45.01 | 26.60±0.66 | 0.55-1.53 | 0.99±0.00 |
|  | Itajaí | 253 | 0.10-46.53 | 13.06±0.58 | 4.79-35.40 | 20.94±0.37 | 5.90-43.22 | 26.19±0.47 | 0.23-1.57 | 1.01±0.00 |

**Table S2** Amplitude, mean (x̄), standard error of burrow density along March 2017 to February 2018 on the beaches of Barra Velha, Navegantes and Itajaí

| Season | Beach | Burrow density (m²) |  |
| --- | --- | --- | --- |
|  |  | Min-Max x̄ |  |
| Spring | Barra Velha | 0.18-0.46 0.15 |  |
|  | Navegantes | 0.25-0.25 0.00 |  |
|  | Itajaí | 0.11-0.35 0.12 |  |
| Summer | Barra Velha | 0.31-0.49 0.12 |  |
|  | Navegantes | 0.25-0.28 0.01 |  |
|  | Itajaí | 0.13-0.29 0.06 |  |
| Autumn | Barra Velha | 0.14-0.40 0.13 |  |
|  | Navegantes | 0.13-0.40 0.07 |  |
|  | Itajaí | 0.14-0.35 0.10 |  |
| Winter | Barra Velha | 0.19-0.50 0.16 |  |
|  | Navegantes | 0.18-0.25 0.03 |  |
|  | Itajaí | 0.15-0.25 0.05 |  |

**Figure S1** Pairwise correlation matrix among morphological and population descriptors of *Ocypode quadrata*: biomass, carapace length, carapace width, and relative condition factor (Kn). Diagonal panels show the density distribution of each variable. Lower panels show scatterplots of pairwise relationships. Upper panels show Pearson correlation coefficients (r), with asterisks indicating statistical significance (**p* < 0.05, ***p* < 0.01, ****p* < 0.001)
